# A temperature-induced hysteretic behavior of resistivity and magnetoresistance of electrodeposited bismuth microbridges for X-ray transition-edge sensor absorbers

**DOI:** 10.1038/s41598-025-27049-y

**Published:** 2025-11-23

**Authors:** Orlando Quaranta, Nunzia Coppola, Lisa Gades, Alice Galdi, Tejas Guruswamy, Ludovico Montella, Alessandro Mauro, Luigi Maritato, Antonino Miceli, Sergio Pagano, Carlo Barone

**Affiliations:** 1 Argonne National Laboratory, 9700 S Cass Ave, Lemont, IL 60439 USA; 2 Dipartimento di Fisica “E.R. Caianiello”, Università degli Studi di Salerno, Via Giovanni Paolo II 132, 84084 Fisciano, SA Italy; 3 Dipartimento di Ingegneria Industriale, Università degli Studi di Salerno, Via Giovanni Paolo II 132, 84084 Fisciano, SA Italy; 4 INFN Gruppo Collegato di Salerno, Università degli Studi di Salerno, 84084 Fisciano, SA Italy; 5 CNR-SPIN Salerno, c/o Università degli Studi di Salerno, 84084 Fisciano, SA Italy

**Keywords:** Materials science, Nanoscience and technology, Physics

## Abstract

This study investigates the temperature-induced hysteretic behavior of resistivity and magnetoresistance in electrodeposited bismuth films, with a focus on their application as absorbers in transition-edge sensors (TESs) for X-ray detection. Through a series of resistivity versus temperature measurements from room temperature to a few Kelvin, we explore the change in the conductive behavior of bismuth electrodeposited on various substrates. Our findings show for the first time both hysteretic and irreversible changes in resistivity as a function of temperature. Further, magnetoresistance measurements reveal notable variations in resistance behavior under different magnetic fields, highlighting the impact of magnetic fields on these films’ electronic transport properties, with an indication of potential weak anti-localization effects at the lowest temperatures. This study not only provides a deeper understanding of bismuth’s conductivity characteristics at low temperatures but also sheds light on the practical implications for developing more effective TESs for synchrotron X-ray facilities.

**Supplementary Information:**

The online version contains supplementary material available at 10.1038/s41598-025-27049-y.

## Introduction

Bismuth is a semimetal recognized for its remarkable physical, electrical, and chemical properties^1,2^. These unique attributes facilitate its use in various applications, such as battery anodes^3^ and superconductors^4^. Moreover, bismuth’s high hydrogen evolution overpotential enhances current efficiency in reductive processes within electrochemical devices, and it exhibits strong electrocatalytic activity for CO_2_ reduction^5^. Additionally, bismuth is an effective material for radiation shielding^6,7^ and has high magnetoresistance^8^, making it suitable for applications in radiation safety and magnetic sensing. In the past decade, a newer and somewhat unusual application of bismuth has emerged in the field of superconducting X-ray detectors.

In recent years, synchrotron X-ray facilities have greatly benefited from the use of transition-edge sensors (TESs) due to their superior energy resolution compared to silicon-drift diode sensors^9^. TESs enable new science in areas such as X-ray emission spectroscopy^10–12^, Compton scattering^13–15^, and X-ray Absorption Fine Structure (XAFS) spectroscopy^16^. One of the primary challenges in developing X-ray TESs is to fabricate an absorber material with the necessary X-ray stopping power, while maintaining good thermalization properties and energy resolution by controlling the total heat capacity^17^. In this regard, a promising combination of materials is gold (Au) and bismuth (Bi) due to their complementary properties. Both materials have a high atomic number (Z), which guarantees effective X-ray absorption. While Au is characterized by a well-known high thermal conductivity and relatively high specific heat^18^, even at cryogenic temperatures, Bi is characterized by a very low specific heat at these temperatures, due to its semimetal nature, which strongly limits the number of available carriers in the conduction band^19^. This makes Bi very useful as an X-ray absorbing material for TESs when in combination with Au.

The typical design of a TES for X-rays has a superconducting thermometer sitting on a suspended Silicon Nitride (SiN) membrane together with a photon absorber, in a so called “sidecar” design, as shown in more detail in the last figure of the Methods section (Fig. 6c). The incidence of a photon in the absorber causes a temporary increase in the temperature of the device, which is monitored in time by the superconducting thermometer, from which the photon energy is measured. This assumes that the photon energy is effectively “instantaneously” converted into heat after absorption and that the absorber is isothermal at any moment in time while its temperature evolution is probed by the superconducting thermometer. Eventually the heat generated by the photon is dissipated in the cryostat, which represents a thermal bath, through the SiN membrane.

The first assumption is justified by the difference between the timescale of the energy down conversion of the photon absorption into thermal phonons (fs to ps) and that of the detector response (ms). The second assumption is valid only if the internal thermal conductance of the absorber is much larger than that of the SiN membrane. When the photon hits the absorber, it induces a local increase in temperature (after the entire energy down conversion into thermal phonons), which spreads across the absorber on a timescale determined by its internal thermal conductance. Eventually the heat reaches the superconducting thermometer on one side of the absorber and is measured. In the end, all the heat generated by the photon absorption is dissipated in the cryostat through the SiN membrane, resetting the detector for a new event. If the internal thermal conductance of the absorber is lower or comparable to that of the SiN membrane, the heat developed in the absorber could start to leak into the thermal bath before reaching the superconducting thermometer. This is especially true when the photon hits the far end of the absorber with respect to the thermometer. This scenario creates a phenomenon called position dependent response. Because of the different way in which the heat can disperse within the absorber and toward the superconducting thermometer and the thermal bath, the same photon can generate a different response in the thermometer and therefore a different estimation of the photon energy. This reduces the photon energy resolution achievable by the detector. The Au layer is crucial to minimize this effect, while the Bi allows the formation of thick absorbers thus still limiting the total heat capacity. The same semimetal nature of the Bi could represent a limiting factor for its thermal conductivity at cryogenic temperatures. Although, in principle, it is possible to estimate the thermal conductivity of the Bi at cryogenic temperatures by measuring its resistivity at those temperatures (a standard approach for metals), this approach can prove challenging due to the way in which this material is typically deposited when used for TES absorbers. Currently, the standard approach for the Bi deposition in TESs is via chemical electroplating on top of an existing sputtered Au layer^20^. This approach guarantees that the Bi does not introduce artifacts in the measured X-ray spectra, typical of devices with Bi deposited via thermal evaporation^21^ (the original approach). The difference in behavior is reflective of the difference in morphological structure of the two types of films. Electrodeposited films are characterized by order of magnitude larger crystals and consequently fewer grain boundaries^21,22^. The presence of an underlying Au layer makes the measurements of the Bi conduction properties challenging, especially at cryogenic temperatures, where the Au conductivity dominates. To overcome this problem, a set of specifically designed 4-wire measurement devices has been developed (from here referred to as microbridges). These structures allow measurement of the resistivity of electrodeposited Bi while removing the contribution from the underlying Au layer. Therefore, it is possible to obtain a more accurate understanding of the electrothermal features of this material combination, which is crucial for optimizing TES performance^23^. In particular, the realized devices showed how the dependence of electrical conductivity on temperature of these electrodeposited Bi films is potentially indicative of diverse conduction phenomena such as weak anti-localization and large magnetoresistance. A better understanding of such multifaceted electrical conductivity is crucial to inform their usage as absorbers for X-rays and to predict the ultimate energy resolving capabilities of TES sensors with Bi absorbers.

In this work, a series of electric and magnetic measurements at temperatures ranging from room temperature to a few Kelvin are described. The investigations have been performed on multiple devices fabricated on different substrates: Sapphire and Hi-Resistivity Silicon. Also, the measurements were performed in two different cryostats: an Adiabatic Demagnetization Refrigerator (ADR) and a cold finger cryocooler, to demonstrate the validity of the data presented. Potential explanations for the phenomena seen are consequently discussed.

## Results

### Resistivity vs. temperature

**Fig. 1:**
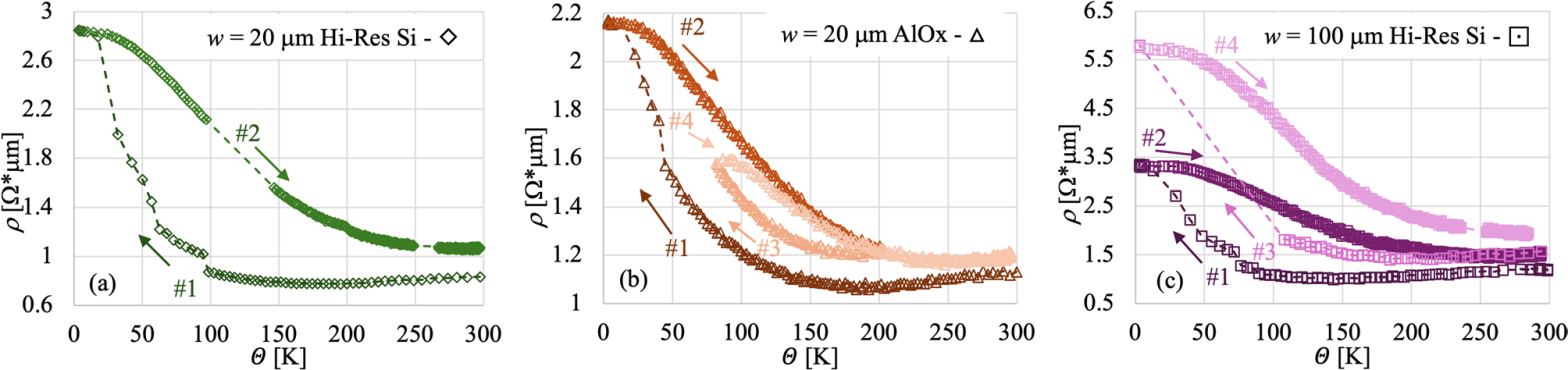
Resistivity versus temperature ($\:\rho\:\left(\varTheta\:\right)$) characteristic for pristine devices of width (**a**) *w* = 20 μm (green diamonds) fabricated on high-resistivity silicon, of width (**b**) *w* = 20 μm (orange triangles) fabricated on sapphire, and of width (**c**) *w* = 100 μm (violet squares) fabricated on high-resistivity silicon. For each device, measurements collected both cooling down and warming up are presented. The arrows indicate the temperature direction and the numbers the temporal sequence (in order from darker to lighter). For the *w* = 100 μm device on Hi-Res Si and for the *w* = 20 μm device on AlOx, two consecutive sets are present.

A series of resistivity versus temperature measurements ($\:\rho\:\left(\varTheta\:\right)$) have been collected on several devices fabricated on a variety of substrates and in multiple instruments. The results are all consistent with each other. Each resistivity datapoint is representative of either an average of 5 consecutive measurements or an integrated measure over several seconds collected at fixed temperatures, depending on whether the resistivity was measured in DC or AC (more details on this are given in the Methods section). To estimate the error on the single resistivity measurement, we calculated the standard deviation of the resistivity for measurements collected at the same temperature assuming 0.1 K of temperature resolution; this came out be $\:\varDelta\:\rho\:\left(\varTheta\:\right)$ ~ 1e−3 Ω µm. Examples of such measurements for microbridges of various widths (*w*), same length (*l* = 100 μm), deposited on different kind of substrates, are shown in Fig. 1, where:

- $\:w$ = 20 μm on Hi-Resistivity Si (Hi-Res), green diamonds—panel (a).
- $\:w$ = 20 μm on Sapphire (AlOx), orange triangles—panel (b).
- $\:w$ = 100 μm on Hi-Resistivity Si, (Hi-Res), violet squares—panel (c).

The Bi average thicknesses ($\:t$) varied from 30 to 40 μm, although the thickness measurement is only indicative due to the high surface roughness caused by the large average grain size (µm)^20^. The sputtered Au thickness for all devices was 100 nm over an adhesion layer of Ti of ~ 5 nm. Starting from room temperature, the resistivity exhibits a metallic temperature dependence (from here referred to as *metallic*), with a small reduction of the resistivity from 300 K down to about 150 K. At this temperature the resistivity starts to increase in a semiconductor-like form (from here referred to as *semiconductive*), although the dependence is not exactly exponential in temperature. This trend is then followed by a saturation of the resistivity at temperatures below ~ 30 K until the base temperature of ~ 3 K (from here referred to as *plateau*). Tests run on an Adiabatic Demagnetization Refrigerator (ADR) cryostat allowed us to see that the *plateau* is maintained all the way down to ~ 0.05 K, the operational temperature of TESs. The consistency of the plateau allowed us to also conduct magnetoresistance measurements, despite the limited cryogenic temperatures (~ 10 K) reachable by a second cryostat, Janis CCS-300 S model (discussed later in the paper). Therefore, we concentrate the analysis only in the range from ~ 300 K down to ~ 10 K. Similar results have been observed in various works involving both single crystal and polycrystalline films as well as patterned devices^24,25^.

The resistivity also presents a series of irreversible vertical jumps, mostly during the cooldown phase. After reaching the base temperature, the resistivity was measured while warming up the sample. The trajectory of the resistivity proved to be similar in shape to the trajectory on the way down, but not identical. The three dependencies (*metallic*, *semiconductive* and *plateau*) are still present, but they develop at different temperatures. Moreover, the slope of the *semiconductive* phase is generally shallower on the way up, effectively reducing the extension of the *metallic* phase. The ending resistivity at room temperature is slightly higher than where it started. The resistivity is thus characterized by a combination of hysteretic behavior and irreversible status changes with temperature, on a scale of an order of magnitude larger than the uncertainty on the single resistivity measure.

To study this phenomenon, measurements over multiple cooldowns have been performed. In Fig. 1 two series of measurements (down and up in temperature) for the Hi-Res Si *w* = 20 μm and *w* = 100 μm devices and the AlOx *w* = 20 μm device are shown. The temperature cycle directions are indicated by arrows and numbers that indicate the temporal sequence #1 first cooldown, #2 fist warmup, #3 second cooldown and #4 second warmup (from darker to lighter). All the devices are pristine, not subject to annealing (more on this later). From these it is possible to see how the general dependencies in both temperature directions stay the same, but the overall resistivity rises a little at every cooldown. Moreover, the specific trajectory of the resistivity in temperature is dependent on the thermal history. The increase in resistivity seems to slow down after multiple cooldowns, and the large irreversible jumps seem to eventually disappear.

The resistivity measurements in the ADR were collected while the temperature was changing freely over several hours (at least 20 h per tempearture cycle, each way), while the resistivity measurements in the cold finger cryocooler were collected while stabilizing the sample at fixed temperature points with a temperature stability of at least 0.1 K. This has been done to rule out eventual thermalization issues with the samples. No variability of the resistivity at a fixed temperature has been identified beyond standard deviation, well below the hysteretic behavior shown in Fig. 1.

The large gaps in the data over tens of Kelvin are caused by purposely introduced interruptions in the measurements aimed to check for consistency. If the jumps in the resistivity were caused by artifacts in the measurement due to interrupting and restarting the acquisition, which requires a long period of warming and cooling for the cryostat, we would expect discontinuities in the data sets. The absence of such discontinuities is indicative of the internal coherence of the data. Also, the fact that the jumps in resistivity are always in the same direction (increase) is indicative of a real physical effect and not simply statistical noise.

To better understand the nature of these results, a set of reference devices have been fabricated. These consisted of long continuous microwires for 4-wire measurements composed of sputtered Au in some cases and of both sputtered Au and electrodeposited Bi in others. Both sets of devices were fabricated on the same wafer and were processed in the same way except for the absence of the bus current patches in Au only devices. As expected, the conductivity in the Au/Bi devices was dominated by the Au layer. By measuring both Au and Au/Bi devices at the same time it was possible to subtract one $\:\rho\:\left(\varTheta\:\right)$ from the other and obtain only the contribution of the Bi. This was characterized by the same trends in the resistivity shown in Fig. 1 but without, or much reduced, hysteresis nor large jumps in resistivity. This demonstrates that these multiphase $\:\rho\:\left(\varTheta\:\right)$ are intrinsic to the electrodeposited Bi and not due to the specific device design used here, while the hysteresis and the jumps seem to be related to the specific microbridge structure. Here, it is important to stress the fact that in order to be useful for further studies, the Au would need to be made quite a bit thinner to reduce its contribution.

To minimize the effect of the thermal history of the samples on the resistivity, i.e. to minimize the number of the large resistivity jumps shown in Fig. 1, the samples were thermally cycled at least 10 times by submerging them in a bath of liquid Nitrogen and bringing them back to room temperature. The measurements discussed below were all done after this treatment.

**Fig. 2:**
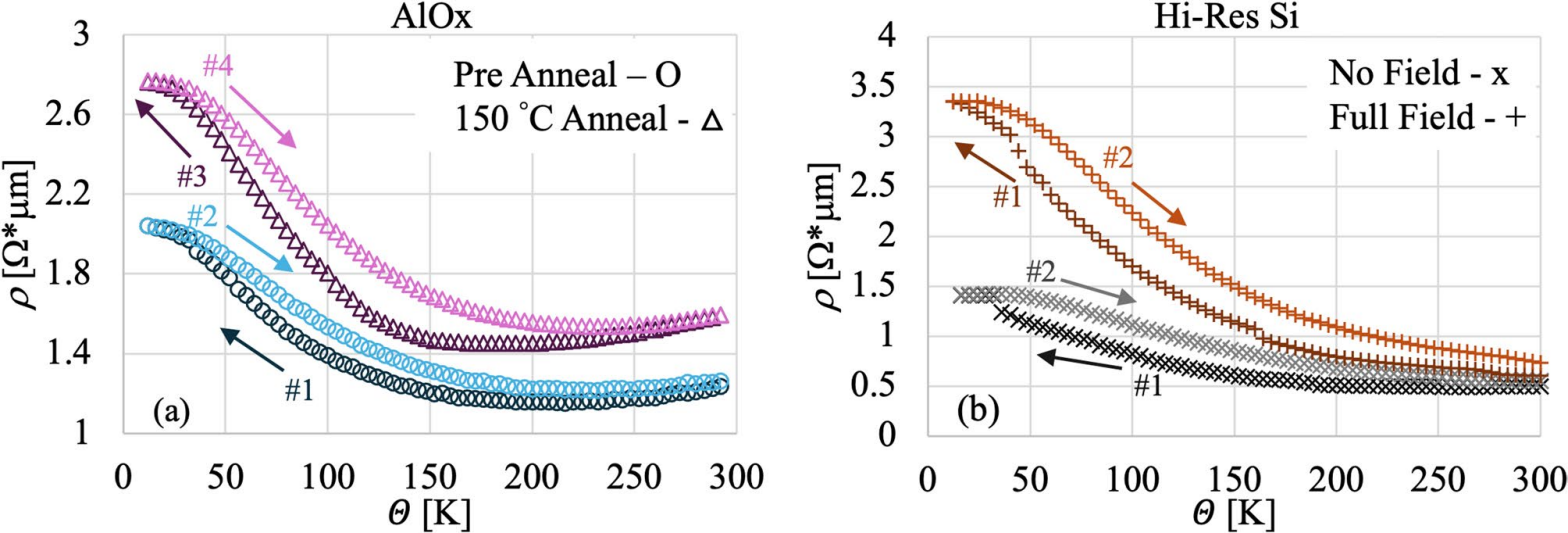
(**a**) Resistivity versus temperature ($\:\rho\:\left(\varTheta\:\right)$) for an AlOx *w* = 20 μm sample. In blue circles are the data for the pristine device, while in violet triangles are the data after annealing at 150 °C for approximately 8 h in an Ar environment. (**b**) Resistivity versus temperature ($\:\rho\:\left(\varTheta\:\right)$) for a Hi-Res Si *w* = 20 μm sample in a perpendicular magnetic field (*B*). In gray exes are the data in zero magnetic field, while in orange pluses are the data in a perpendicular magnetic field of 0.77 T. The arrows indicate the temperature direction and the numbers the temporal sequence (in order from darker to lighter).

To study the role of the film morphology, the *w* = 20 μm width on AlOx sample was annealed in an Argon atmosphere for about 8 h at 150 °C. The resistivity in temperature was measured before and after the annealing, by cooling it down to about 10 K and then subsequently warming it up to 300 K. The results are presented in Fig. 2a, where, similarly to Fig. 1, the temperature cycle directions are indicated by arrows and the numbers indicate the temporal sequence.

Although the general trend is the same both before (blue circles) and after annealing (violet triangles), the details are different. Both data sets show the typical hysteretic behavior, but there are clear differences. Contrary to what one could expect^25^, there is an overall increase in the resistivity and a change in the shape. After annealing, the extension of the *metallic* part of the resistivity is increased, extending all the way down to about 150 K. This is followed by the typical *semiconductive* increase but at a much steeper rate. Finally, the resistivity reaches the *plateau* below ~ 30 K. Another difference is in the size of the hysteresis in temperature, notably large in the sample after annealing, especially between $\:T$ = 75 K and $\:T$ = 200 K, reflective of the major difference in the extension of the *metallic* phase. Moreover, the hysteresis in resistivity at room temperature is smaller after annealing.

### Magnetoresistance vs. temperature

To better understand the nature of the conductivity phenomena that result in the resistivity versus temperature seen in the previous section, a series of magnetoresistance ($\:MR\left(B,\varTheta\:\right)$) measurements have been performed. The samples were mounted in a cryostat that allowed for the measurement of resistance while a variable magnetic field ($\:B$) orthogonal to the sample surface was applied. The magnetoresistance, defined as the percentage variation of the resistance in field with respect to the resistance at zero field $\:MR\left(B,\varTheta\:\right)=\frac{\left[R\left(B,\varTheta\:\right)-R\left(0,\varTheta\:\right)\right]}{R\left(0,\varTheta\:\right)}$, was measured on multiple samples at various temperatures. The measurements covered the various conductivity regimes in temperature and were performed both during cooldown and warmup. The magnetic field was varied between two extremes: − 0.77 T to 0.77 T. The cryostat allowed for the stabilization of the sample temperature with a precision of at least 0.1 K and the measurements were conducted by sweeping the magnetic field between the two extremes multiple times. The overall acquisition per temperature point lasted tens of minutes, during which the resistance at zero field always returned to the original value, proving that the hysteresis in resistance shown before cannot be ascribed to poor sample thermalization.

For reference, here we will discuss the magnetoresistance results for the Hi-Res Si *w* = 20 μm pristine sample; similar results have been obtained for the other pristine samples. The magnetic field increases the overall resistance of the device at all temperatures, but it also changes the distribution of the various phases. At full field, the *metallic* phase is absent, in favor of a much-extended *semiconductive* phase, and the *plateau* is greatly reduced, as shown in Fig. 2b.

**Fig. 3:**
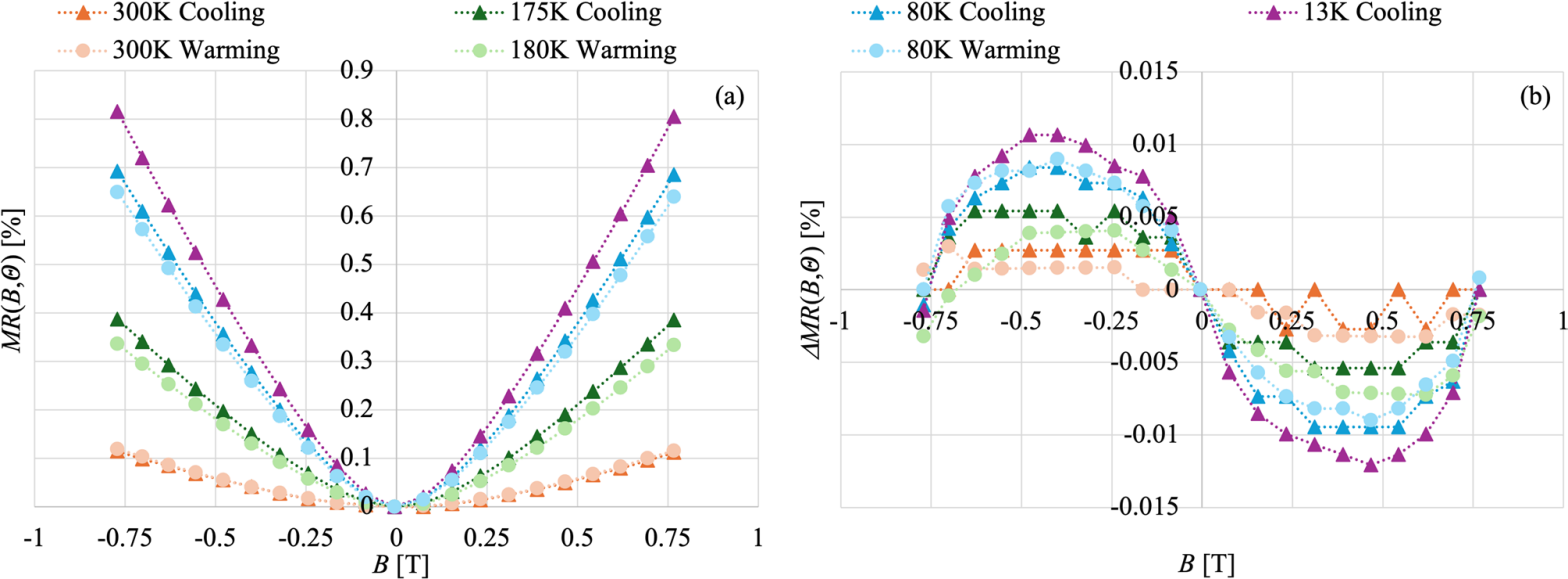
(**a**) Magnetoresistance ($\:MR\left(B,\varTheta\:\right)$) of a Hi-Res Si *w* = 20 mm pristine sample measured at various temperatures ($\:\varTheta\:$) both during cooldown (triangles) and warmup (circles). Data for each of the three phases of the resistivity in temperature curve are present: 300 K—*metallic*, 180 K and 80 K—*semiconductive*, 13 K—*plateau*. (**b**) Hysteresis in the magnetoresistance ($\:{\Delta\:}MR\left(B,\varTheta\:\right)$) with respect to the direction of the magnetic field (*B*) sweep—from − 0.77 T to 0.77 T and vice versa. The scattering of the data at 300 K is reflective of the limited resolution on the resistance measurements and not a physical effect.

The magnetoresistance was measured at fixed temperatures (stability of at least 0.1 K) that cover the entire range of conductivity behavior seen in the previous section: 300 K—*metallic*, 180 K and 80 K—*semiconductive*, 13 K *plateau* (Fig. 3a), both cooling down and warming up. The resistance has a strong dependence on the magnetic field, even at these low fields. Similar trends have been seen in other works^23,24^. The data sets referring to cooling down and warming up share the same dependence in temperature, but not the same absolute values of $\:MR\left(B,\varTheta\:\right)$, especially at higher fields. Moreover, the sample presents a small magnetic hysteresis ($\:\varDelta\:MR\left(B,\varTheta\:\right)={MR}_{forw}-{MR}_{back}$). When the magnetic field is swapped from one extreme to another ($\:{MR}_{forw}$ measured from 0.77 T to − 0.77 T) and back ($\:{MR}_{back}$ measured from − 0.77 T to 0.77 T), the $\:MR\left(B,\varTheta\:\right)$ does not follow exactly the original trace, but it is slightly higher or lower depending on the sign of the field. This hysteresis grows with the lowering of the temperature, similarly to the $\:MR\left(B,\varTheta\:\right)$ behavior but on a much smaller scale, as shown in Fig. 3b (the scattering of the data at 300 K is reflective of the limited resolution on the resistance measurements and not a physical effect). This hysteresis cannot be ascribed only to the inherent hysteresis of the electromagnet used in the experiments, which is much lower than what is seen here and has a dependence on the electromagnet bias current very different from what we see in our samples—maximum 0.01 T (refer to Methods sections for more details).

**Fig. 4:**
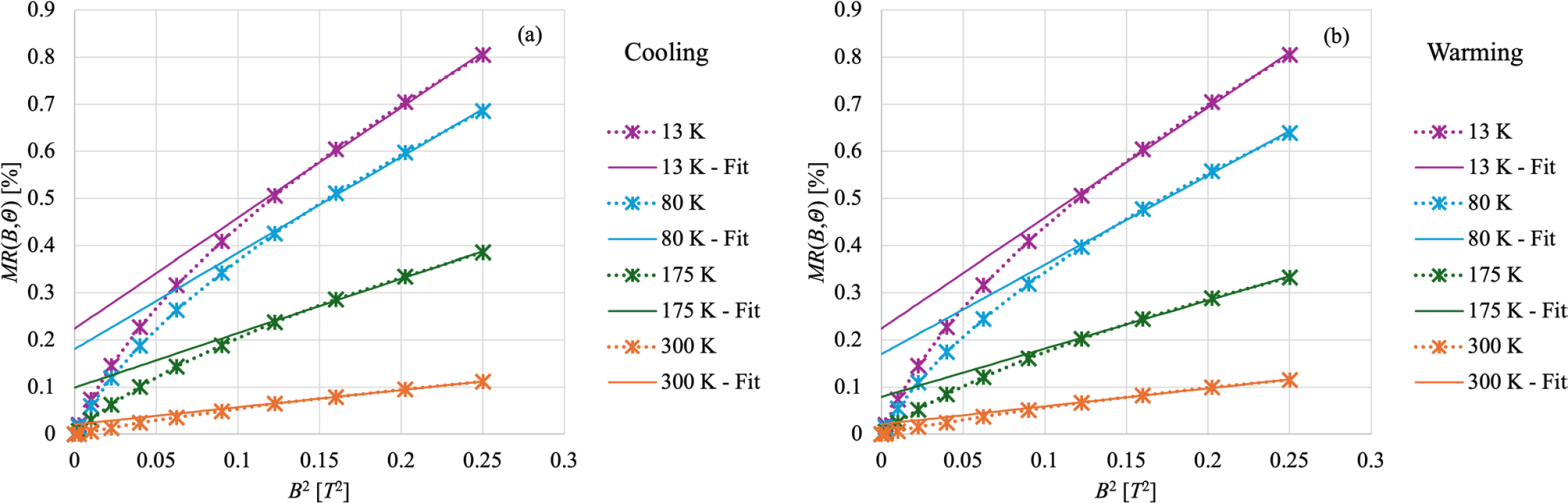
Magnetoresistance ($\:MR\left(B,\varTheta\:\right)$) as a function of magnetic field squared (*B*^2^) at various temperatures, measured during cooldown (**a**) and warmup (**b**). In both, data sets are represented with stars and dashed lines. Solid lines, instead, are the fits to a linear dependence of $\:MR\left(B,\varTheta\:\right)$ on $\:{B}^{2}$ using only the higher field points.

Finally, the dependence of the $\:MR\left(B,\varTheta\:\right)$ on the applied magnetic field is not the same at all temperatures. Classical magnetoresistance theory predicts a quadratic dependence at low field, followed by a saturation at high fields^26^. In Fig. 4 is represented the $\:MR\left(B,\varTheta\:\right)$ as a function of the magnetic field intensity squared ($\:{B}^{2}$) in stars and dashed lines measured at various temperatures, both while cooling down (a) and while warming up (b). In the same figure are also shown the fits to a linear dependence of $\:MR\left(B,\varTheta\:\right)$ on $\:{B}^{2}$ using only the higher field points in solid lines. If the typical quadratic dependence was maintained, the data should lie on a straight line for all values of $\:{B}^{2}$. Figure 4 shows how the data follow a $\:{B}^{2}$ dependence only towards the higher fields. Moreover, the further down in temperature the more the dependence moves away from $\:{B}^{2}$, with data at the lowest temperatures having a steeper dependence on $\:B$. Similar behavior has been observed in other works^27,28^ although at higher fields and lower temperatures.

## Discussion

The conduction properties of Bi films have been the subject of extensive studies throughout the years, both in continuous films^27^ and in patterned structures^28^. Bi wires have been widely investigated due to the unique electronic properties of this element^29,30^. Being a semimetal with a very small indirect band-overlap (38 meV at 0 K), Bi has a temperature-dependent electron density $\:{n}_{e}$ (3 × 10^18^ cm^−3^ at 300 K, 3 × 10^17^ cm^−3^ at 4 K), which is four to five orders of magnitude lower than that of ordinary metals. The Fermi surface of Bi consists of ellipsoidal pockets for electrons and holes, resulting in small effective masses $\:{m}_{eff}\:$(as low as 0.001 $\:{m}_{e}$—electron mass $\:{m}_{e}$) and large Fermi wavelengths $\:{\lambda\:}_{F}$ (∼40 nm)^31^. Moreover, mean free paths $\:{l}_{e}$ are as long as ∼100 nm at 300 K and ∼400 μm at 4 K. These characteristics, together with the phonon scattering, cause the mobility to depend on temperature. A non-monotonic resistivity over temperature behavior can occur, which is usually attributed to an additional limitation of the mean free path by temperature independent boundary scattering^30,31^.

The resistivity as a function of temperature curves shown in Fig. 1 are common in microstructures of electrodeposited Bi^25^ and can be partially explained by the interplay between the temperature dependence of the electrical carrier density and the electron mean free path. In semimetals, the Fermi temperature may be of the order of 100 K^32^, which results in a limited carrier density at low temperatures with a notable temperature dependence. At the same time, the mean free path increases with decreasing temperature and can be very long in Bi at cryogenic temperatures. These two competing effects can explain the peculiar shape of the $\:\rho\:\left(\varTheta\:\right)$, that at room temperature starts with a metallic behavior, as in a regular metal, where $\:{l}_{e}$ increases with a decreasing temperature, while $\:{n}_{e}$ remains essentially constant. As the temperature decreases, approaching the Fermi temperature, $\:{n}_{e}$ will start to reduce. When this reduction becomes faster than the increase in $\:{l}_{e}$, and when $\:{l}_{e}$ becomes limited by the average dimension of the grain, the resistivity stops behaving like a metal (where $\:{n}_{e}$ would remain essentially constant) and starts to behave like a semiconductor, with an increase in resistivity. This increase is not precisely semiconductive in nature. In a semiconductor/insulator, the electron density of states has an exponential dependence on temperature $\:{n}_{e}\left(\varTheta\:\right)\propto\:{e}^{\frac{{E}_{G}}{2{k}_{B}\varTheta\:}}$, where $\:{E}_{G}$ is the energy gap and $\:{k}_{B}$ is the Boltzmann constant, which would generate a corresponding exponential dependence in $\:\rho\:\left(\varTheta\:\right)$, not present in our data. Further reducing the temperature generates a plateau in the resistivity. This could (partially) be explained by the finite $\:{n}_{e}$ at these temperatures (typical of semi-metal, unlike semiconductors) and by the saturation of $\:{l}_{e}$ induced by the average crystal dimension. The presence of surface metallic states, which can dominate the conductivity at these temperatures, can also explain the saturation effect of the resistivity.

Although these phenomena have been seen in multiple works on Bi microstructures (films and wire), the data presented in this work are characterized by some unique fingerprints. In particular, the $\:\rho\:\left(\varTheta\:\right)$ curves show the presence of irreversible jumps in the resistivity, which tend to be more common during the first cooldowns and tend to disappear after several thermal cycles. These jumps in $\:\rho\:$ could be explained by mechanical reallocation of the crystals in the microwire, especially in the areas where the various crystals of Bi growing on top of Au surfaces merge through lateral growth in the areas where the Au is not present, typical of these microbridge devices. These changes are due to a coefficient of thermal expansion in Bi several times higher than that of the substrates and of the Au^32^; such mismatch between the film and the substrate could lead to mechanical stress when devices are cooled. The faster contraction of the Bi crystals with respect to the substrate could increase the spacing between crystals in the microwire, with a consequent increase in the potential energy between grains, which would increase the measured resistivity. Conversely, this hypothesis does not explain why the modification should be permanent. Another hypothesis is the formation of defects in the single crystals during the cooldowns. These defects can effectively reduce the average dimension of the crystals, further limiting the electron mean free path at cryogenic temperatures. This would cause an irreversible sudden increase in resistivity, as seen in our data. We also tried to anneal our sample at ~ 150 °C for 8 h in an atmosphere of Argon (Ar). The resistivity overall increased, but the *metallic* phase extended to lower temperatures (Fig. 2a). This partially differs from what other groups have seen^24^, where they have observed a notable reduction in the resistivity, together with a general change in the shape of the $\:\rho\:\left(\varTheta\:\right)$. In the referred work^24^, the original electrodeposited samples show $\:\rho\:\left(\varTheta\:\right)$ very similar to the ones reported in Fig. 1, while after a suitable annealing of 268 °C for 6 h in Ar, the polycrystalline films become single crystals with the trigonal axis orientated perpendicular to the film plane. In these annealed films, the *metallic* phase greatly extends, essentially eliminating the *semiconductive* and the *plateau* sections. The effect is explained in terms of reduction (or effective disappearance) of grain boundary scattering. In our devices, performing the annealing at a much lower temperature (150 °C) appears to have not allowed for a complete reformation of the crystallographic structure of the film, even though these temperatures were expected to be sufficient^33^.

A possible explanation for the overall increase in the resistivity could be the inelastic deformation of the film, caused by the relatively high annealing temperature without any photolithographic mold to constrain the shape (the annealing was performed on the complete device), which could have reduced the cross section in the area between the two voltage leads. We have also tried to identify obvious signs of such deformations via Scanning Electron Microscope imaging of a representative device before and after an annealing at 150 °C for 8 h. The images, presented in Fig. 2 of the supplementary information, don’t show obvious differences in the Bi crystal shapes. This doesn’t necessarily rule out the possibility of morphological deformations in the device, especially at the interface between the two sides of the Bi growth, which could be too subtle to the see via this approach. On the other hand, the image after the anneal shows traces migration of Bi into the Au leads, which suggests that there is ionic mobility at 150 °C and this can affect the grain boundaries. Such migration effect has also been reported in reference^34^, where they also partially ascribed the measured changed in conduction properties of the bilayer to the interdiffusion between the two metals. Similar effects could explain the increased extension of the metallic phase. Dedicated experiments will be necessary to more accurately verify this hypothesis.

Ultimately, the objective of this anneal was to test the stability of the film in real processing conditions that an electrodeposited Bi film could encounter during the fabrication of a TES. During the fabrication of TESs for X-ray, annealing at 150 °C of the devices can be used to tune the device critical temperature, by affecting the quality of the films used in the bilayer that constitute the thermometer element of the device (typically Mo and Au or Mo and Cu) and the quality of their interface^35^. In this sense, this test proves that one must be very careful when performing these annealings because they can negatively affect the thermal conductivity of the Bi absorbers.

Another peculiarity present in our data, not reported elsewhere (to the best of our knowledge), is the hysteretic behavior of $\:\rho\:\left(\varTheta\:\right)$. The general shape is the same both cooling down and warming up, with all the various phases present: *metallic*, *semiconductive* and *plateau*, but with different extension. This indicates that the conduction phenomena are the same in direction but influenced by some hysteretic effect. This cannot be due to poor thermalization of the devices under test because the data were collected either over several hours (~ 20 h cycle) in both directions or by thermalizing the sample at a fixed temperature point via a PID-controlled sample stage. The effect could be symptomatic of permanent deformations in the morphological configuration of the disordered crystals in the microbridge (Fig. 6a,b), due to the differential thermal contraction between the Bi and the substrate. Bi is characterized by an anisotropic coefficient of thermal expansion (CTE), which can be approximated to an average ~ 15 × 10^− 6^ K^-1^ at 300 K for polycrystalline films, which decreases linearly at lower temperatures^36^. Conversely, crystalline Si has a CTE of ~ 3 × 10^− 6^ K^−1^ which also decreases linearly in temperature but changes sign below 120 K^37^. Under this hypothesis, mechanical deformations of the crystal configuration can negatively impact the overall film conduction properties. Similarly in the case of AlOx substrates, these have a CTE ~ 6 × 10^− 6^ K^−1^ at 300 K (again quite different from Bi) which reduces very quickly at cryogenic temperatures, with approximately a *T* ^*3*^ dependence^38^. Furthermore, the same behavior was seen in devices fabricated on amorphous SiO_2_ over Si wafers, with a CTE ~ 0.55 × 10^− 6^ K^−1^ at 300 K^39^, as shown in Fig. 1 of the supplementary information. Moreover, the presence of macroscopic voids in the Bi film, clearly visible in Fig. 6b, can further affect the electrical and thermal conduction properties of the film when deformed by the stress induced by the different CTEs. The morphological configuration in electrodeposited Bi films can vary greatly, depending on the deposition conditions. Both the grain dimensions and the compactness of the film can be tuned by switching from DC to pulsed deposition and by changing the duty cycle^40^.

This represents a point of concern when these Bi films are used in TESs, where they are deposited on suspended SiN membranes of about 500 nm thickness. These membranes, once released, tend to bow to adapt to the mechanical constraints introduced by the various thick metallic layers deposited on top of them, with consequent impact on the Bi conductivity in the TES absorber.

Another hypothesis (not necessarily exclusive to the previous one) is that the peculiar behavior of the resistivity could be due to some kind of magnetic effect. Bi is known to be a material characterized by strong magnetic field dependent electrical conduction properties: from giant magnetoresistance^24^, to Spin-Orbit Kondo effects^41^, to weak anti-localization^28^. Our measurements do indeed show a reasonably strong magnetoresistance (Fig. 4), although smaller than what has been measured in single crystal Bi or in annealed electrodeposited Bi films^24,25^. In Fig. 2b we can see how the $\:\rho\:\left(\varTheta\:\right)$ is strongly influenced by the applied orthogonal magnetic field. Not only does the overall resistance increase, but also the shape of the $\:\rho\:\left(\varTheta\:\right)$ changes. In particular, the magnetic field completely removes the *metallic* phase at the temperatures of the experiment in favor of the *semiconductive* phase. This is the same result shown by other groups and can be explained by the interplay between the magnetoresistance and the strong increase in conductor mean path in Bi at cryogenic temperatures^24,25^. Even at full field, the $\:\rho\:\left(\varTheta\:\right)$ is hysteretic in temperature, similarly to what we have seen at zero field. The $\:MR\left(B,\varTheta\:\right)$ measurement also shows a hysteresis in the magnetic field (Fig. 3b), which disappears at zero fields, thus excluding effects of residual magnetization. This large $\:MR\left(B,\varTheta\:\right)$ represents an added reason for being extremely careful with respect to residual magnetic fields when working with TESs.

Another interesting aspect of the $\:MR\left(B,\varTheta\:\right)$ in these films is that it not only grows with reducing temperatures, as expected due to the increase in the conductor mean free path, but it also changes shape. The lower the temperature the further the $\:MR\left(B,\varTheta\:\right)$ dependence on $\:B$ is from the standard $\:{B}^{2}$ dependence, as shown by the quality of the quadratic fits shown in Fig. 4. At base temperature the $\:MR\left(B,\varTheta\:\right)$ shows a pronounced dip at low $\:B$, typical of weak anti-localization. In disordered two-dimensional systems, electrons - due to their wave-like nature - can interfere with themselves. This interference can double the probability of an electron returning to its starting point, which typically suppresses the conductance, a phenomenon called weak localization^42–44^. However, when strong spin-orbit coupling is present, the electron spins rotate in opposite directions, leading to destructive interference, which consequently increases the conductance, a phenomenon called weak anti-localization^45,46^. Weak anti-localization is usually challenging to identify via magnetoresistance measurements because the effect can be overshadowed by the large classical magnetoresistance. Typically to achieve a clear indication of weak anti-localization very thin films are needed, which strongly reduce the electron mean free path and consequently the classical magnetoresistance effect. Moreover, the effect is more evident at extreme cryogenic temperatures. Weak anti-localization effects have been shown in Bi thin films^28^. Our experimental setup, used for magneto-transport measurements, does not allow us to reach temperatures below ~ 13 K and our samples are intrinsically thicker and composed of larger grains than those studied in Ref.^28^, but nonetheless in our $\:MR\left(B,\varTheta\:\right)$ measurements there are hints of this effect. The same weak anti-localization effect could explain the presence of the plateau at low temperatures.

The overall increase in resistivity of the Bi both with decreasing temperatures and increasing magnetic field has a direct effect on the thermal proprieties of Bi. The thermal conductivity in metals is linked to the material resistivity via the phenomenological law of Wiedemann–Franz $$\:\kappa\:=\sigma\:*L*\varTheta\:$$, where $$\:\kappa\:$$ is the thermal conductivity, $$\:\sigma\:$$ is the electrical conductivity ($$\:\sigma\:\left(\varTheta\:\right)=1/\rho\:\left(\varTheta\:\right))$$, $$\:L$$ is the Lorenz number and $$\:\varTheta\:$$ is temperature. The applicability of this law to semi-metal is only partially valid, but it can still provide information on the order of magnitude of the thermal conductivity of the material, enough for a comparative analysis with the other materials present in a TES^23^. Considering a pristine sample, starting from room temperature and zero magnetic field, the expected thermal conductivity is $$\:\kappa\:$$ ~ 5 W m^−1^ K^−1^ (averaged among the various devices). Cooling the Bi to cryogenic temperatures, especially below 150 K, causes a large decrease in the thermal conductivity, which decreases more than an order of magnitude to $$\:\kappa\:$$ ~ 0.11 W m^−1^ K^−1^ at 12 K. This value is limited by the plateau in resistivity, without which the thermal conductivity would reduce even further by at least a factor two, estimated by extrapolating the resistivity following the trend in the *semiconductive* section. In this estimation, the value for bulk Bi was used for the Lorenz number. This assumption is justified by the reasonable agreement between the measured resistivity of our devices with the resistivity of bulk Bi^18^. Similarly, after an annealing in Ar at 150 °C, the permanent increase in resistivity at all temperatures, and in particular at cryogenic temperatures, causes a further and permanent reduction in the Bi thermal conductivity to $$\:\kappa\:$$ ~ 0.08 W m^−1^ K^−1^ at 12 K. Although this anneal was much longer than the typical anneal used to stabilize Mo/Au TESs, it is important to keep in mind the potential effect of annealing on the thermal conductivity of the Bi absorber.

Similarly, in presence of magnetic fields the resistivity increases quickly, especially at low fields and low temperatures, where the magnetoresistance grows faster than $\:{B}^{2}$. At a field of $\:B$ ~ 0.77 T and at 12 K, $\:\rho\:\left(\varTheta\:\right)$ increases by a factor ~ 2.3, consequently the thermal conductivity decreases of the same factor. Moreover, the change in magnetoresistance shows a hysteretic behavior with the sweep of the magnetic field. Although the magnetic fields used here are very large when referred to the use of Bi as absorber for X-ray TESs, and would destroy the superconductivity in the thermometer, these considerations still provide another reason for effectively magnetically shielding TESs.

The thermal conductivities at cryogenic temperatures of Bi estimated here are more than two orders of magnitude lower than that of Au (~ 7.8 W m^−1^ K^−1^). This could indicate potential position dependence problems when used as part of an X-ray absorber in a TES, depending on how it compares to the other thermal conductivities in the device, or more precisely, how all the thermal conductances compare. We estimated the thermal conductivity for our SiN membranes at cryogenic temperature to be ~ 1.2 × 10^− 3^ W m^−1^ K^−1^, extracted from the current-voltage characteristics of our devices^17^. This is orders of magnitude lower than that the thermal conductivity of both Au and Bi. To estimate the actual thermal conductance in the absorber, let’s assume that the photon is absorbed on the surface of the absorber. After the initial energy down-conversion process, which generates a cloud of secondary electrons, the energy dissipates in the form of thermal phonons. Assuming an area from which the phonons start to diffuse downwards in the absorber equal to the radius of the secondary electron cloud and considering the total thickness of the absorber as the total length that the phonon needs to travel, we can estimate the total thermal conductance of the absorber. This number is dependent on the photon energy, both because the thickness of the absorber is typically tuned to achieve a good stopping efficiency at a given photon energy, and because the radius of the secondary electron cloud depends on the photon energy. Assuming a photon of 12 keV, the secondary electron cloud has a radius of ~ 100 nm^22^. The typical Bi thickness for these sensors is 20 μm. The estimated thermal conductance for the Bi is ~ 0.15 nW/K, which is the same order of magnitude of the typical thermal conductance of the SiN membrane for these devices^17^. This sets a lower limit to the actual thermal conductance of Bi. In reality, the phonon will not just move vertically down within the footprint of the secondary electron cloud but will spread in all directions, effectively increasing the total thermal conductance. Nonetheless, this approximation shows how having limited thermal conductivity in the Bi can potentially represent a problem for its use in these detectors. If, for example, the thickness grows to 100 μm, a minimum thickness for 100 keV photons, the thermal conductance can drop another order of magnitude. At this point, obvious signs of position dependence could start to manifest.

Currently, only devices composed of Bi layers up to 20 μm have been routinely tested, both by us and other groups^47,48^. These do not show obvious signs of position dependence, in accordance with this discussion. This condition might not be respected for much thicker layers of Bi, though. Transition Edge Sensors for photons of energy greater than 100 keV currently make use of absorbers made of macroscopic blocks of Tin (Sn) manually glued on top of the membrane. This approach poses a limit to the size of the pixel arrays that can be realistically fabricated. An approach based on the growth of very thick Bi films via electrodeposition would greatly simplify the problem, allowing for the growth of thick Bi absorbers for large arrays of TES. To be successful, this approach would need to keep under control the increase in electrical resistivity (and equivalently the decrease in thermal conductivity) of the electrodeposited Bi films, expecially if negatively affected by mechanical stress in the film induced by the cryogenic temperatures. To minimize these effects, different electrodeposition conditions can be explored, as shown in reference^40^ where they demonstrate how the use of AC pulsed currents can affect film morphology. However, more studies are needed to completely explore the complex parameter space of the deposition settings (amplitude, sign and duration of the pulses, duty cycle, etc.). Alternatively, a post-deposition annealing can increase the average grain size with consequent reduction in the film RRR^34^, but this process is only compatible with certain types of TES based on alloys (e.g. AlMn), rather than typical bilayer TESs (e.g. MoAu or MoCu).

## Conclusion

In this work, the electrical and magnetic conduction properties of electrodeposited Bi microbridges have been studied under various bias, temperature and magnetic field conditions. The data provide valuable information towards the understanding of the conduction properties at sub-K temperatures in electrodeposited Bi, crucial to the design of effective TESs for X-rays. The typical large grain structure present in electrodeposited Bi films, although it represents a strong advantage when used as absorbing material in TESs for X-rays, is more susceptible to magnetic fields, especially at cryogenic temperatures where a strong magnetoresistance dependence to low fields is present. The increased resistivity could affect the thermalization properties of the absorber, hence particular care needs to be used in shielding these devices from stray magnetic fields.

Further studies focused on quantum effects at very low temperatures, like weak anti-localization and Kondo, would be useful. In particular, employing experimental investigations on 1/f noise spectroscopy^49,50^ could provide important information regarding the physical mechanisms in action^43,44,51,52^. Finally, in-situ X-ray diffraction experiments at temperatures between 300 K and 3 K performed at synchrotron facilities could give more direct insight into the crystals morphology and how they change in temperature. All these experiments could shine a brighter light on Bi conduction properties at these temperatures, which is of great interest to the single photon detector community.

## Methods

### Sample preparation

**Fig. 5:**
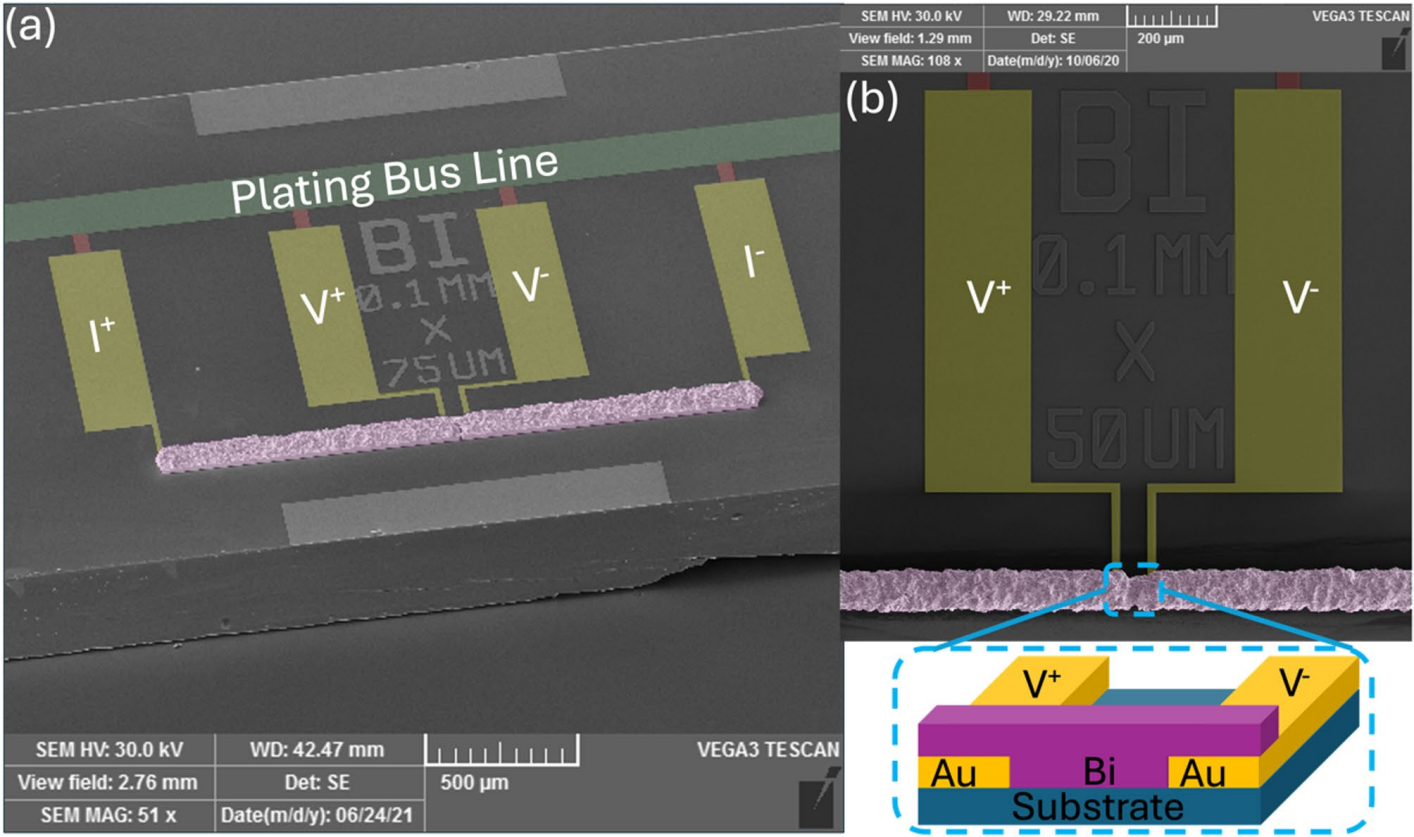
(**a**) Scanning Electron Microscope (SEM) picture of a representative device. In yellow are visible the pads for both the bias and the voltage measurements. The pads are made of sputtered Au. In violet is shown the Bi microbridge under study. Under the Bi is a layer of Au (not visible) that acts as seed layer for Bi plating. In green is represented the bus line used for the Bi plating, and in red are the connection patches to the bus line, which are removed at the end of the fabrication process. (**b**) A close-up view and a schematic representation of the Bi microbridge. The conductivity of Bi is measured between the two voltage contacts where only Bi is present (no Au seed layer).

The devices used in these experiments have been discussed extensively elsewhere^23^, but their nature can be summarized as following. The devices are typical 4-wire resistivity measurement structures, built around a microwire structure, represented in Fig. 5.

The Bi film is grown via electroplating on top of a sputtered Au seed layer^20^. The 4-wire structure is achieved via a series of masks that define the shape of the various layers: a lift-off mask for the Au seed layer, together with the plating bus line and the connection patches, and a plating mask for the Bi microbridge. The Bi mask is shaped to allow the growth of the Bi from the Au lines, which also act as seed layers. The growth of the Bi in the plating solution is isotropic in every direction, therefore it will not only grow vertically but also horizontally, within the boundaries of the mold defined by the lithography. Thus, the Bi growing from the Au leads will meet in the area between the leads, over the bare substrate. This creates a microbridge between the Au lines composed only of Bi, without any Au underneath. This guarantees that measured properties are relative to the Bi and not influenced by the Au seed layer. The devices were fabricated on a two different type of substrates: high-resistivity silicon (Hi-Res Si) and sapphire (AlOx), both insulators, so that the electrical measurements are not influenced by the substrate. In these structures, the two external contacts serve as the input and output lines for the bias current, and the two internal contacts are used to measure the voltage drop across the microbridge.

**Fig. 6:**
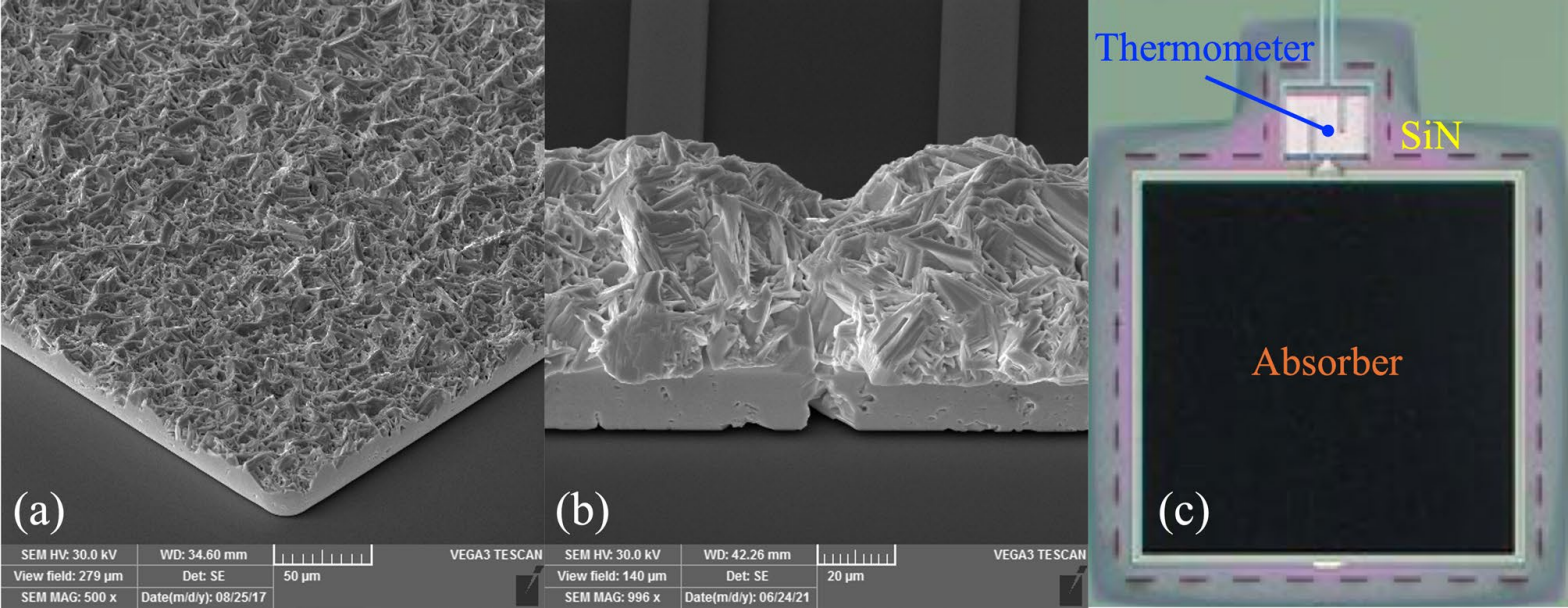
(**a**) Scanning Electron Microscope (SEM) picture of a representative Bi film as an X-ray absorber for TESs. (**b**) Scanning Electron Microscope (SEM) picture of a representative Bi microbridge for conductivity measurements. (**c**) Optical microscope picture of a Transition Edge Sensor. Highlighted are the photon absorber, the superconducting thermometer and the SiN membrane.

The microscopic structure of the Bi in the microbridge appears identical to the typical structure found in electrodeposited Bi layers used as X-ray absorbers in TESs. An example of both type of structures is shown in Fig. 6.

### Electric and magneto-transport measurement setup

The zero magnetic field DC electrical transport measurements were performed in a cryogen free Adiabatic Demagnetization Refrigerator (ADR) from FormFactor, equipped with a variable temperature stage capable of operating between 300 K and 50 mK. The sample temperature was measured with a RuOx resistor thermometer, in contact with the sample holder. The samples were characterized via the use of a Stanford Research Systems SIM921 AC resistance bridge which applied a bias of 3 µA, avoiding any potential self-heating effect. The sample temperature was varied over cycles of several hours (at least 20 h each way), allowing time for the complete thermalization of the sample with the sample holder.

**Fig. 7:**
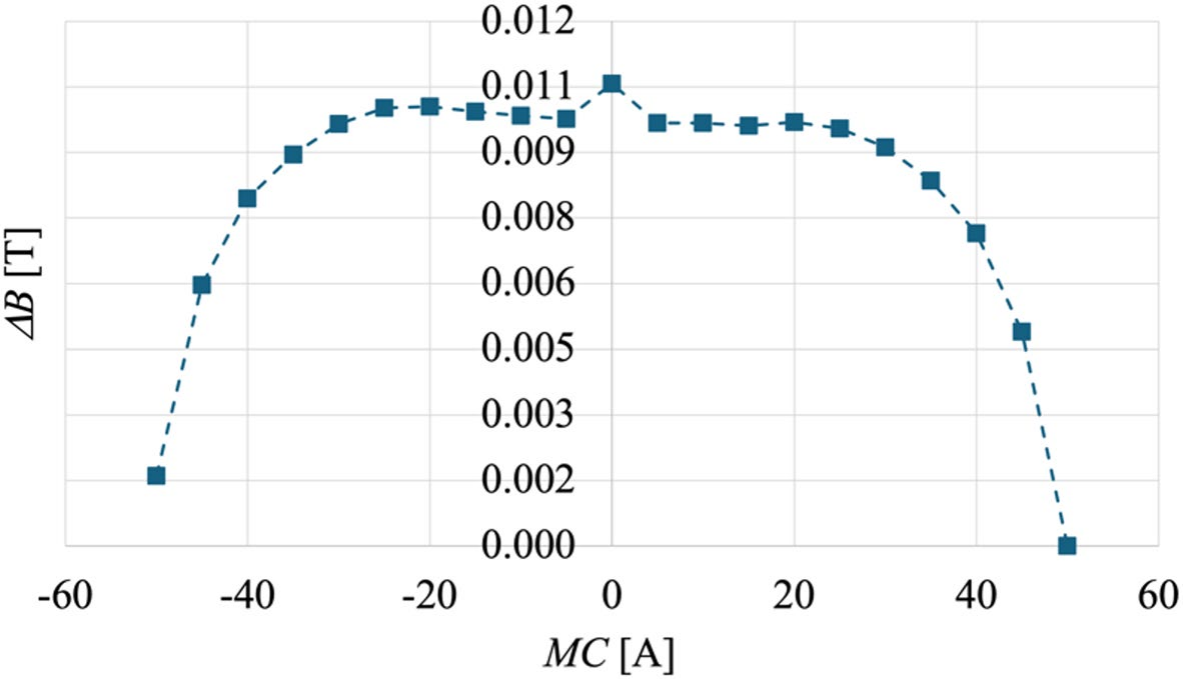
Electromagnet hysteresis expressed in variation of magnetic field, measured by sweeping the bias of the electromagnet first in one direction and then in the opposite.

The magnetic field transport measurements were carried out on a Janis CCS-300 S cold finger cryocooler (Janis Research Company, Inc, USA) in a temperature range from 325 K to 10 K. This system is a closed cycle refrigerator based on the Gifford-McMahon thermodynamic cycle coupled with a single stage water cooled He gas compressor (Sumitomo HC-4E1 Helium Compressor, Sumitomo (SHI) Cryogenics of America, Inc., USA). The temperature stabilization, realized through a computer-controlled feedback loop, was better than 0.01 K. Magnetic characterization is performed by coupling the cryostat with an electromagnet (Lake Shore Cryotronics Model EM4-HVA, Lakeshore Inc., USA) with induced magnetic fields up to ± 0.77 T. $\:MR\left(B,\varTheta\:\right)$ curves were acquired by sending currents ranging from 0 to ± 50 A to the magnet (where ± stands for the current orientation) with 5 A steps; the induced magnetic field intensity is measured with an Hall Probe (425 Gaussmeter, Lake Shore Cryotronics, Inc). The measured maximum hysteresis in the magnetic field ($\:\varDelta\:B$) is less than 0.01 T throughout the entire electromagnet bias current range ($\:MC$), as seen in Fig. 7.

## Supplementary Information

Below is the link to the electronic supplementary material.

Supplementary Material 1

## Data Availability

The data that support the findings of this study are available from the corresponding authors upon reasonable request.

## References

[1] Sandnes, E., Williams, M. E., Bertocci, U., Vaudin, M. D. & Stafford, G. R. Electrodeposition of bismuth from nitric acid electrolyte. *Electrochim. Acta*. **52**, 6221–6228. 10.1016/j.electacta.2007.04.002 (2007).

[2] Yang, M. & Hu, Z. Electrodeposition of bismuth onto glassy carbon electrodes from nitrate solutions. *J. Electroanal. Chem.* **583**, 46–55. 10.1016/j.jelechem.2005.04.019 (2005).

[3] Zhang, R., Bao, J., Wang, Y. & Sun, C. Concentrated electrolytes stabilize bismuth-potassium batteries. *Chem. Sci.* **9**, 6193–6198. 10.1039/c8sc01848k (2018). 30090306 10.1039/c8sc01848k PMC6062896

[4] Sato, K. et al. Bismuth superconducting wires and their applications. *Cryogenics* **33** (3), 243–246. 10.1016/0011-2275(93)90038-P (1993).

[5] Abdallah, R. et al. New porous bismuth electrode material with high surface area. *J. Electroanal. Chem.* **839**, 32–38. 10.1016/j.jelechem.2019.03.023 (2019).

[6] Elsafi, M., El-Nahal, M. A., Sayyed, M. I. & Abbas, M. I. Novel 3-D printed radiation shielding materials embedded with bulk and nanoparticles of bismuth. *Sci. Rep.* **12**, 12467. 10.1038/s41598-022-16317-w (2022). 35864112 10.1038/s41598-022-16317-w PMC9304356

[7] Wang, B., Ting, C., Lai, C. & Tsai, Y. Bismuth pelvic X-ray shielding reduced radiation dose exposure in pediatric radiography. *Biomed. Res. Int.* **2021**, 9985714. 10.1155/2021/9985714 (2021). 10.1155/2021/9985714 PMC8523245 34671681

[8] Jiang, S., Huang, Y., Luo, F., Du, N. & Yan, C. Synthesis of bismuth with various morphologies by electrodeposition. *Inorg. Chem. Commun.* **6**, 781–785. 10.1016/S1387-7003(03)00104-7 (2003).

[9] Heald, S. M. Strategies and limitations for fluorescence detection of XAFS at high flux beamlines. *J. Synchrot Radiat.* **22**, 436–445. 10.1107/S1600577515001320 (2015). 10.1107/S1600577515001320 PMC4786056 25723945

[10] Guruswamy, T., Gades, L., Miceli, A., Patel, U. & Quaranta, O. Beamline spectroscopy of integrated circuits with hard X-ray transition edge sensors at the advanced photon source. *IEEE Trans. Appl. Super*. **31**, 2101605. 10.1109/TASC.2021.3067246 (2021).

[11] Guruswamy, T. et al. Hard X-ray fluorescence measurements with TESs at the advanced photon source. *J. Phys. Conf. Ser.* **1559**, 012018. 10.1088/1742-6596/1559/1/012018 (2020).

[12] Lee, S. et al. Soft X-ray spectroscopy with transition-edge sensors at Stanford synchrotron radiation lightsource beamline 10 – 1. *Rev. Sci. Instrum.* **90**, 113101. 10.1063/1.5119155 (2019). 31779391 10.1063/1.5119155

[13] Patel, U. et al. Development of transition-edge sensor X-ray microcalorimeter linear array for Compton scattering and energy dispersive diffraction imaging. *J. Low Temp. Phys.* **199**, 384–392. 10.1007/s10909-019-02267-7 (2020).

[14] Kothalawala, V. N. et al. Extracting the electronic structure of light elements in bulk materials through a Compton scattering method in the readily accessible hard X-ray regime. *Appl. Phys. Lett.* **124**, 223501. 10.1063/5.0207375 (2024).

[15] Patel, U. et al. High-resolution Compton spectroscopy using X-ray microcalorimeters. *Rev. Sci. Instrum.* **93**, 113105. 10.1063/5.0092693 (2022). 36461526 10.1063/5.0092693

[16] Holt, M., Harder, R., Winarski, R. & Rose, V. Nanoscale Hard X-Ray microscopy methods for materials studies. *Ann. Rev. Mater. Res.* **43**, 183–211. 10.1146/annurev-matsci-071312-121654 (2013).

[17] Irwin, K. D. & Hilton, G. C. *Cryogenic Particle Detection* 63–150 (Springer, 2005). 10.1007/b12169

[18] Dean, J. A. *Lange’s Handbook of Chemistry 15th edn.* (McGraw-Hill, 1999). 10.4236/ns.2014.61003

[19] Collan, H. K., Krusius, M. & Pickett, G. R. Specific heat of antimony and bismuth between 0.03 and 0.8 K. *Phys. Rev. B*. **1**, 2888. 10.1103/PhysRevB.1.2888 (1970).

[20] Gades, L. et al. Development of Thick electroplated bismuth absorbers for large collection area hard X-ray transition edge sensors. *IEEE Trans. Appl. Super*. **27**, 2101105. 10.1109/TASC.2017.2662007 (2017).

[21] Yan, D. et al. Eliminating the non-Gaussian spectral response of X-ray absorbers for transition-edge sensors. *Appl. Phys. Lett.* **111**, 192602. 10.1063/1.5001198 (2017).

[22] Yan, D. et al. Microstructure analysis of bismuth absorbers for transition-edge sensor X-ray microcalorimeters. *J. Low Temp. Phys.* **193**, 225–230. 10.1007/s10909-018-1888-1 (2018).

[23] Quaranta, O. et al. Devices for thermal conductivity measurements of electroplated Bi for X-ray TES absorbers. *J. Low Temp. Phys.* **209**, 1165–1171. 10.1007/s10909-022-02876-9 (2022).

[24] Yang, F. Y. et al. Large magnetoresistance of electrodeposited single-crystal bismuth thin films. *Sci. Rep.* **284**, 1335–1337. 10.1126/science.284.5418.1335 (1999). 10.1126/science.284.5418.1335 10334983

[25] Yang, F. Y. et al. Large magnetoresistance and finite-size effect in electrodeposited bismuth lines. *J. Appl. Phys.* **89**, 7206–7208. 10.1063/1.1357115 (2001).

[26] Pippard, A. B. *Magnetoresistance in Metals*. 10.1126/science.245.4920.874.a (Cambridge University Press, 1989). 10.1126/science.245.4920.874 17773367

[27] Yin, S. L., Liang, X. J. & Zhao, H. W. Effect of a highly metallic surface state on the magneto-transport properties of single crystal Bi films. *Chin. Phys. Lett.* **30**, 087305. 10.1088/0256-307X/30/8/087305 (2013).

[28] Sangiao, S. et al. Quantitative analysis of the weak anti-localization effect in ultrathin bismuth films. *EPL* **95**, 37002. 10.1209/0295-5075/95/37002 (2011).

[29] Heremans, J. et al. Bismuth nanowire arrays: synthesis and galvanomagnetic properties. *Phys. Rev. B*. **61**, 2921. 10.1103/PhysRevB.61.2921 (2000).

[30] Chiu, P. & Shih, I. A study of the size effect on the temperature-dependent resistivity of bismuth nanowires with rectangular cross-sections. *Nanotechnology* **15**, 1489. 10.1088/0957-4484/15/11/020 (2004).

[31] Lin, Y. M., Sun, X. & Dresselhaus, M. S. Theoretical investigation of thermoelectric transport properties of cylindrical Bi nanowires. *Phys. Rev. B*. **62**, 4610. 10.1103/PhysRevB.62.4610 (2000).

[32] Brown, P. *High-pressure states of bismuth*, PhD Thesis, University of Cambridge (2017). 10.17863/CAM.16819

[33] Oksanen, M. *Annealing Study of Bismuth Thin Films*, Master Thesis, University of Jyvaskyla (2010).

[34] Zhang, L. et al. Annealing-induced conductivity enhancement of electroplated bismuth for transition-edge sensors. *IEEE Trans. Appl. Super*. 10.1109/TASC.2025.3606868 (2025).

[35] Weber, J. C. Development of a transition-edge sensor bilayer process providing new modalities for critical temperature control. *Supercond. Sci. Technol.* **33**, 115002. 10.1088/1361-6668/abb206 (2020).

[36] White, G. K. Thermal expansion of trigonal elements at low temperatures: As, Sb and Bi. *J. Phys. C Solid State Phys.* **5**, 2731. 10.1088/0022-3719/5/19/006 (1972).

[37] Lyon, K. G., Salinger, G. L., Swenson, C. A. & White, G. K. Linear thermal expansion measurements on silicon from 6 to 340 K. *J. Appl. Phys.* **48**, 865–868. 10.1063/1.323747 (1977).

[38] Colin, T. et al. Measurement of the thermal expansion coefficient of an all-sapphire optical cavity. *IEEE Trans. Instr. Meas.* **46**, 183–185. 10.1109/19.571807 (1997).

[39] Bansal, N. P. & Doremus, R. H. *Handbook of Glass Properties*. 10.1016/C2009-0-21785-5 (Academic Press, Science Direct, 1986).

[40] Hatfield, K. O. et al. Electrodeposition and analysis of thick bismuth films. *Sci. Rep.* **13**, 1202. 10.1038/s41598-023-28042-z (2023). 36681686 10.1038/s41598-023-28042-z PMC9867696

[41] Craco, L. & Leoni, S. Magnetoresistance in the spin-orbit Kondo state of elemental bismuth. *Sci. Rep.* **5**, 13772. 10.1038/srep13772 (2015). 26358556 10.1038/srep13772 PMC4566097

[42] Bergmann, G. Weak localization in thin films: a time-of-flight experiment with conduction electrons. *Phys. Rep.* **107**, 1–58. 10.1016/0370-1573(84)90103-0 (1984).

[43] Barone, C. et al. Universal origin of unconventional 1/ f noise in the weak-localization regime. *Phys. Rev. B*. **87**, 245113. 10.1103/PhysRevB.87.245113 (2013).

[44] Barone, C. et al. Nonequilibrium fluctuations as a distinctive feature of weak localization. *Sci. Rep.* **5**, 10705. 10.1038/srep10705 (2015). 26024506 10.1038/srep10705 PMC4448654

[45] Lee, P. A. & Ramakrishnan, T. V. Disordered electronic systems. *Rev. Mod. Phys.* **57**, 287. 10.1103/RevModPhys.57.287 (1985).

[46] Bergmann, G. Weak Anti-localization - An experimental proof for the destructive interference of rotated spin ½. *Solid State Commun.* **42**, 815–817. 10.1016/0038-1098(82)90013-8 (1982).

[47] Guruswamy, G., Quaranta, O., Gades, L. & Miceli, A. Measurement of subtle X-ray emission line shifts at the advanced photon source with transition edge sensors. *IEEE Trans. Appl. Super*. **35**, 2101605. 10.1109/TASC.2025.3540045 (2025).

[48] Saito, T. Y. et al. Application of hard X-Ray and Gamma-Ray TES microcalorimeter at accelerator facility. *IEEE Trans. Appl. Super*. **35**, 2100805. 10.1109/TASC.2024.3525445 (2025).

[49] Barone, C. & Pagano, S. What can electric noise spectroscopy tell us on the physics of perovskites? *Coatings*. **11**, 96. 10.3390/coatings11010096 (2021).

[50] Diaz, S. A. & Di Ventra, M. The role of measurement time on the universal crossover from 1/f to non-1/f noise behavior. *J. Comput. Electron.* **14**, 203–208. 10.1007/s10825-014-0641-5 (2015).

[51] Barone, C. et al. Kondo-like transport and magnetic field effect of charge carrier fluctuations in granular aluminum oxide thin films. *Sci. Rep.* **8**, 13892. 10.1038/s41598-018-32298-1 (2018). 30224642 10.1038/s41598-018-32298-1 PMC6141613

[52] Barone, C. et al. Current-resistance effects inducing nonlinear fluctuation mechanisms in granular aluminum oxide nanowires. *Nanomaterials* **10**, 524. 10.3390/nano10030524 (2020). 32183260 10.3390/nano10030524 PMC7153260

