# Supplementary material for: A temperature-induced hysteretic behavior of resistivity and magnetoresistance of electrodeposited bismuth microbridges for X-ray transition-edge sensor absorbers
Source: Sci Rep. 2025 Nov 23;15:42969. doi: 10.1038/s41598-025-27049-y (PMC12673135; doi:10.1038/s41598-025-27049-y)
Supplement: Supplementary file 1 — Supplementary Material 1 [file 41598_2025_27049_MOESM1_ESM.docx]

**Supplementary Information:**

**A temperature-induced hysteretic behavior of resistivity and magnetoresistance of electrodeposited bismuth microbridges for X-ray transition-edge sensor absorbers**

Orlando Quaranta^1,2,*^, Nunzia Coppola^3^, Lisa Gades^1^, Alice Galdi^3^, Tejas Guruswamy^1^, Ludovico Montella^1,2^, Alessandro Mauro^2,4^, Luigi Maritato^3^, Antonino Miceli^1^, Sergio Pagano^2,4,5^, and Carlo Barone^2,4,5^

^1^Argonne National Laboratory, 9700 S Cass Ave, Lemont, IL 60439, USA

^2^Dipartimento di Fisica “E.R. Caianiello”, Università degli Studi di Salerno, Via Giovanni Paolo II 132, 84084 Fisciano (SA), Italy

^3^Dipartimento di Ingegneria Industriale, Università degli Studi di Salerno, Via Giovanni Paolo II 132, 84084 Fisciano (SA), Italy

^4^INFN Gruppo Collegato di Salerno, c/o Università degli Studi di Salerno, 84084 Fisciano (SA), Italy

^5^CNR-SPIN Salerno, c/o Università degli Studi di Salerno, 84084 Fisciano (SA), Italy

E-mail: [oquaranta@anl.gov](mailto:oquaranta@anl.gov)


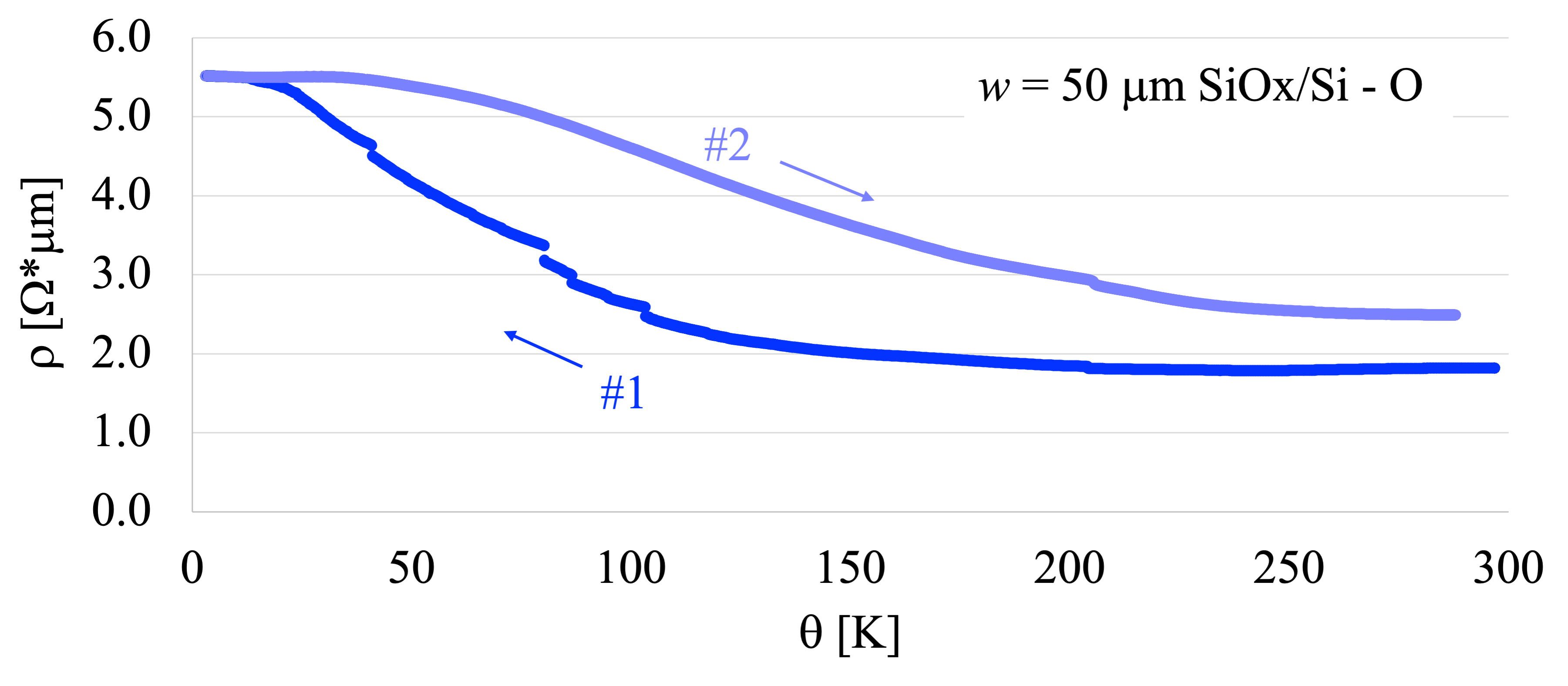


**Supplementary Figure 1 |** **Resistivity versus temperature (**$\boldsymbol{\rho}\boldsymbol{(}\boldsymbol{\Theta}\boldsymbol{)}$**) characteristic for a pristine device of width *w* = 50 μm (blue circles) fabricated on silicon oxide on silicon.**

Measurements collected both cooling down and warming up are presented. The arrows indicate the temperature direction and the numbers the temporal sequence (in order from darker to lighter).


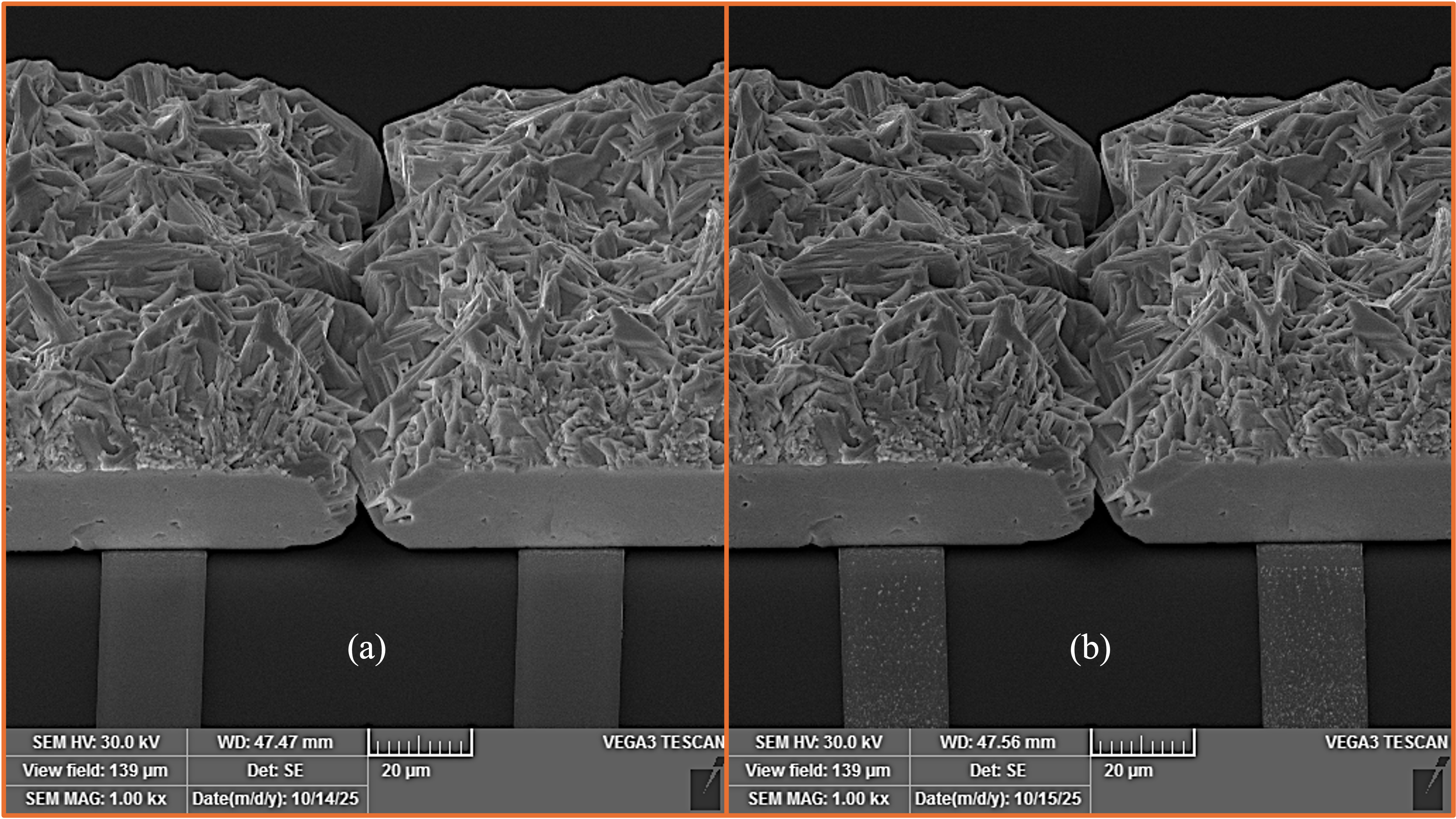


**Supplementary Figure 2 |** **SEM image of a representative device before (a) and after (b) annealing at 150 °C for 8 hours.**

SEM images of a representative device pre- (a) and post-anneal (b) at 150 °C for 8 hours. The morphology of the Bismuth film does not change visibly at this magnification, but more subtle changes, especially at the interface of the two sides of the growth, could still be present. The presence of diffused Bi particles can be seen in the Au leads after the anneal, suggests that there is ionic mobility at 150C and this can affect the grain boundaries.
